# Self-assembly of *Eucalyptus gunnii* wax tubules and pure ß-diketone on HOPG and glass

**DOI:** 10.3762/bjnano.12.70

**Published:** 2021-08-20

**Authors:** Miriam Anna Huth, Axel Huth, Kerstin Koch

**Affiliations:** 1 Rhine-Waal University of Applied Sciences, Faculty of Life Sciences, Marie-Curie-Str. 1, 47533 Kleve, Germany

**Keywords:** ß-diketone tubules, eucalyptus, plant wax, recrystallization, self-assembly

## Abstract

Eucalyptus trees and many plants from the grass family (Poaceae) and the heather family (Ericaceae) have a protective multifunctional wax coating on their surfaces made of branched ß-diketone tubules. ß-diketone tubules have a different size, shape, and chemical composition than the well-described nonacosanol tubules of the superhydrophobic leaves of lotus (*Nelumbo nucifera*). Until now the formation process of ß-diketone tubules is unknown. In this study, extracted wax of *E. gunnii* leaves and pure ß-diketone were recrystallized on two different artificial materials and analyzed by scanning electron microscopy (SEM) and atomic force microscopy (AFM) to study their formation process. Both the wax mixture and pure ß-diketone formed tubules similar to those on *E. gunnii* leaves. Deviating platelet-shaped and layered structures not found on leaves were also formed, especially on areas with high mass accumulation. High-resolution AFM images of recrystallized ß-diketone tubules are presented for the first time. The data showed that ß-diketone tubules are formed by self-assembly and confirmed that ß-diketone is the shape-determining component for this type of tubules.

## Introduction

The plant cuticle, which is the largest biological interface, covers all aerial non-lignified parts of higher plants [1]. It is an extracellular membrane of epidermal cells, consisting of a matrix of the polymer cutin and a mixture of hydrophobic compounds, the cuticular waxes [2–3]. This interface is a multifunctional surface optimized by evolution to protect plants from environmental stress [4]. Waxes are, thereby, essential for a variety of functions, especially in the wettability and self-cleaning ability of plant surfaces [5–6]. Plant waxes consist of a complex mixture of aliphatic and aromatic compounds. The exact chemical composition of the wax varies between plant species and ontogeny of plant organs [7]. Typical wax components are hydrocarbons (C_20_ to C_40_) and derivatives, such as fatty acids, aldehydes, and alcohols. Cuticular waxes can be classified according to their location in intra- and epicuticular waxes. The former is incorporated into the cutin matrix and the latter is deposited on the cutin layer, building the outermost layer of the cuticle. The two types of waxes may also differ in chemical composition [8–9].

Epicuticular waxes form various three-dimensional structures with different sizes (0.5–100 µm) and morphologies [5]. As early as 1871, de Bary proposed the designation “crystal” for the wax structures [10]. This hypothesis was later verified by X-ray diffraction [11–12]. The most common crystalline structure of epicuticular wax crystals is the orthorhombic order [13]. Studies of growing plant waxes showed that wax morphologies are formed by a self-assembly process. On artificial surfaces, waxes recrystallize in three-dimensional structures with similar morphology and related properties as on plant surfaces [14]. Additionally, recrystallization of isolated wax components revealed that usually the main compounds determine the shape of the wax crystals [15–19].

Epicuticular waxes appear in different shapes and the main types are crusts, threads, rodlets, platelets, plates, and tubules [20]. Wax tubules are hollow structures with different sizes and chemical compositions. Depending on their chemical and morphological characteristics, different tubule types were distinguished. Most tubules belong to the group of secondary alcohol tubules, predominantly containing nonacosanol and homologues, or to the group of ß-diketone tubules, containing high amounts of ß-diketones, such as hentriacontane-14,16-dione. Furthermore, alkanediol tubules, characterized by high amounts of alkandiols, and lactone tubules are also described [17,21–24]. Nonacosanol and alkanediol tubules are common in gymnosperms and in some dicotyledonous families, such as Ranunculaceae (buttercup family) and Magnoliaceae (magnolia family) [20]. These tubules have a triclinic crystal order, a length of 0.3–1.1 µm, and a diameter of 0.1–0.2 µm [13,15,17]. ß-Diketone tubules have a similar diameter of 0.1–0.2 µm and a length of 2–3 µm, therefore, they are longer than the other two types of tubules and they have a hexagonal crystal structure [13,18].

Secondary alcohol tubules evolved in all major groups of land plants and design durable superhydrophobic surfaces (e.g., the Lotus Effect). In vitro recrystallization experiments with single wax components of these tubules showed that tubules were formed by secondary alcohols plus at least 2% of diols [25]. The expected arrangements of the tubule-forming molecules are bended layers of nonacosanol and alkanediol. Within these layers, the hydroxyl groups of the alcohol molecules function as spacers in the tightly packed layer, leading to a curvature of the layer and eventually to the formation of the tubules [26]. Atomic force microscopy (AFM) investigations further showed that the elongation of secondary alcohol tubules is based on a helical growth mechanism [27]. Recrystallization experiments with nonacosan-10-ol on non-biological substrates showed that the chemical and physical properties of the underlying substrate affected the crystal formation [27–28].

**Figure 1:**
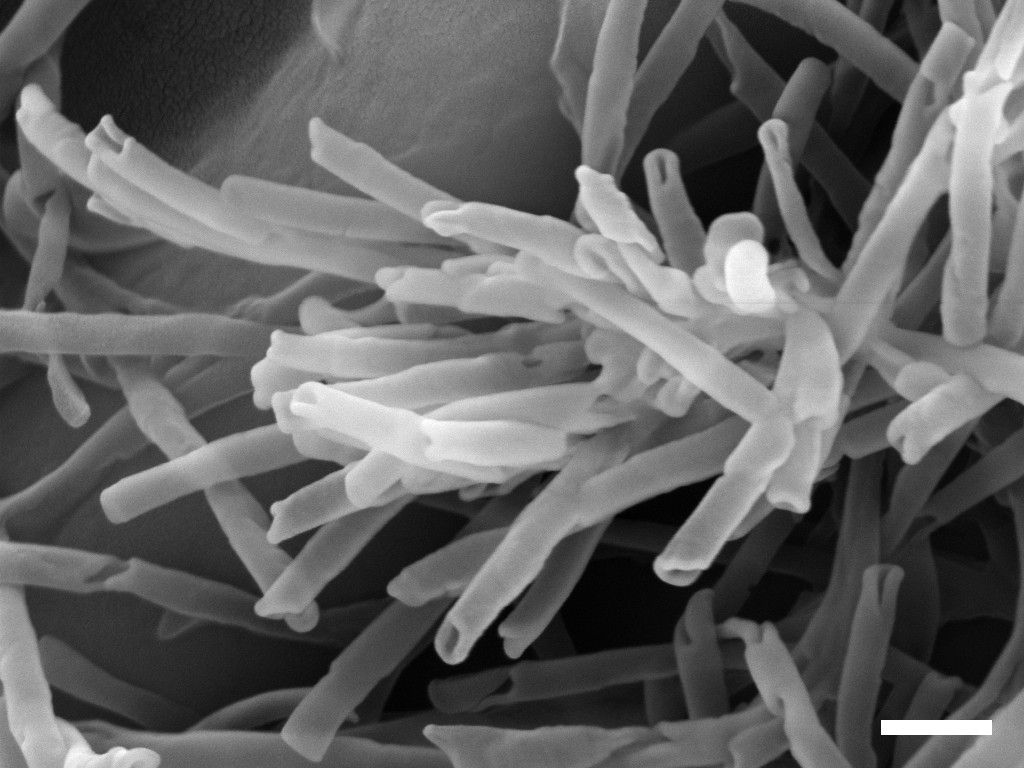
SEM micrograph of ß-diketone tubules on an *E. gunnii* leaf surface. Scale bar 400 nm.

ß-Diketone tubules have been recrystallized by total wax extracts and isolated ß-diketone of plants, which present ß-diketone tubules on their surfaces [18,29]. However, until now, the process of formation of this chemically and morphologically different type of wax tubules is unknown. On *Eucalyptus gunnii* leaves mainly the characteristic ß-diketone tubules are present (Figure 1), but helically wound ribbons and a transitional form between both shapes are also present. Atomic force microscopy investigations of tubule formation on living leaves of *E. gunnii* showed that new tubules were formed after the removal of the wax layer. However, no information about the tubule formation process was obtained [30]. In this study, ß-diketone tubules were recrystallized from extracted *E. gunnii* wax on non-biological surfaces to investigate the tubule formation process by AFM in real time. Additionally, recrystallization experiments with pure ß-diketone were performed to study its role in the formation of the tubules. The major constituents of tubular *E. gunnii* wax are triacontane-14,16-dione (C_30_H_58_O_2_) analyzed by Koch et al. [31], hentriacontane-14,16-dione (C_31_H_60_O_2_) mentioned by Wirthensohn et al. [32–33], and tritriacontane-14,16-dione (C_33_H_64_O_2_) as published by Li et al. [34]. These ß-diketones belong to the most frequent ß-diketones found in plant waxes [35]. Here, extracted wax mixtures of *E. gunnii* leaves and hentriacontane-14,16-dione were used to study their recrystallization by SEM and AFM.

## Experimental

### Substrates used for recrystallization

Recrystallization experiments of a wax solution and a ß-diketone solution were done on two substrates with different physicochemical properties. Glass microscope coverslips were used as polar, amorphous substrates. The glasses were cleaned with chloroform before their use in recrystallization studies. Highly oriented pyrolytic graphite (HOPG) was used as non-polar, crystalline substrates (SPI supplies, West Chester, USA). Freshly cleaned HOPG surfaces were prepared by stripping off a layer of HOPG with double-sided adhesive tape.

### Wax extraction from leaves

Recrystallization experiments were carried out with a 0.1% (w/v) chloroform (99.8%, ACS reagent, Acros, New Jersey, USA) solution of extracted wax of *E. gunnii* and a 0.1% (w/v) chloroform solution of ß-diketone. The wax of *E. gunnii* was extracted from approximately 50 leaves taken from 6–7 month old plants cultivated in a greenhouse for previous experiments. For the extraction, the wax leaves were dipped in chloroform at room temperature for 60 s. The wax composition was analyzed by gas chromatography–flame ionization detection (GC–FID). The wax was mainly composed of the ß-diketone triacontan-14,16-dione and triterpenoids, followed by primary alcohols. Other minor compound classes were secondary alcohols, alkanes, fatty acids, and aldehydes [31]. The used hentriacontan-14,16-dione (97% purity) isolated from barley (*Hordeum vulgare* L.) was analyzed by GC–FID and provided by Prof. P. von Wettstein-Knowles, University of Copenhagen, Denmark. For recrystallization, a 20 µL droplet of the wax solution and of the ß-diketone solution, respectively, were applied to the substrates at room temperature.

### Scanning electron microscopy

The specimens with recrystallized structures of wax and ß-diketone were prepared for SEM (Gemini Supra 40VP, Zeiss, Oberkochen, Germany) three days after the evaporation of the solvent. They were fixed on aluminum stubs (Plano, Wetzlar, Germany) with double-sided adhesive tape (Double-sided Tape Universal, Tesa, Hamburg, Germany) and conductive carbon cement (Leit-C, Plano, Wetzlar, Germany) was applied. The samples were sputter coated (108 auto/SE, Cressington, Watford, UK) with gold (*t*: 60 s, *I*: 30 mA, *p*: 0.1 mbar), resulting in a gold layer of approximately 8 nm. Recrystallized structures were then investigated by SEM (In-lense detector, 5 kV, WD: 7.9–9.1 mm). The procedure was repeated three times per substrate.

### Atomic force microscopy

Real-time observations of recrystallization were performed by consecutive image recording with an atomic force microscope (NanoWizard II, JPK instruments, Berlin, Germany). For this, freshly prepared substrates were fixed on a microscope glass slide by double-sided adhesive tape. The ß-diketone solution was applied to glass substrates and the wax solution was applied to glass and HOPG. The measurements were started directly after the evaporation of the bulk of the solvent and were done in tapping mode with tapping-mode cantilevers (Tap300-G, Budget Sensors, Sofia, Bulgaria). The scan rates ranged from 0.7 to 2.3 Hz and the scan sizes from 3 × 3 to 10 × 10 µm. The maximum possible set point was used (approx. 60–70% of drive amplitude). Obtained topography and amplitude data of AFM measurements were processed and analyzed with JPK data processing software (version 4.2.62). The height of the tubules and the thickness of the layers were investigated using the cross section function of the processing software. The tubule height was calculated by averaging the height differences between the maximum tubule length to the left-side base and to the right-side base of the tubule. The height growth velocity of the tubules was calculated by linear regression lines of the height values as a function of time. The thickness of the layers (*n* = 10) was measured by the mean value of five measurement points. After JPK data processing, the software ImageJ (version 1.51f) was used to measure tubule length.

### Data analysis

All obtained row data were evaluated in Excel (2010, version 14, Microsoft, Redmond, USA). The results are given as mean values ± standard deviation. The graphical representation was done with SigmaPlot (version 13, Systat Software Inc, San Jose, USA).

## Results

### SEM of recrystallized wax and ß-diketone

#### Distribution and morphology of recrystallized structures

**Figure 2:**
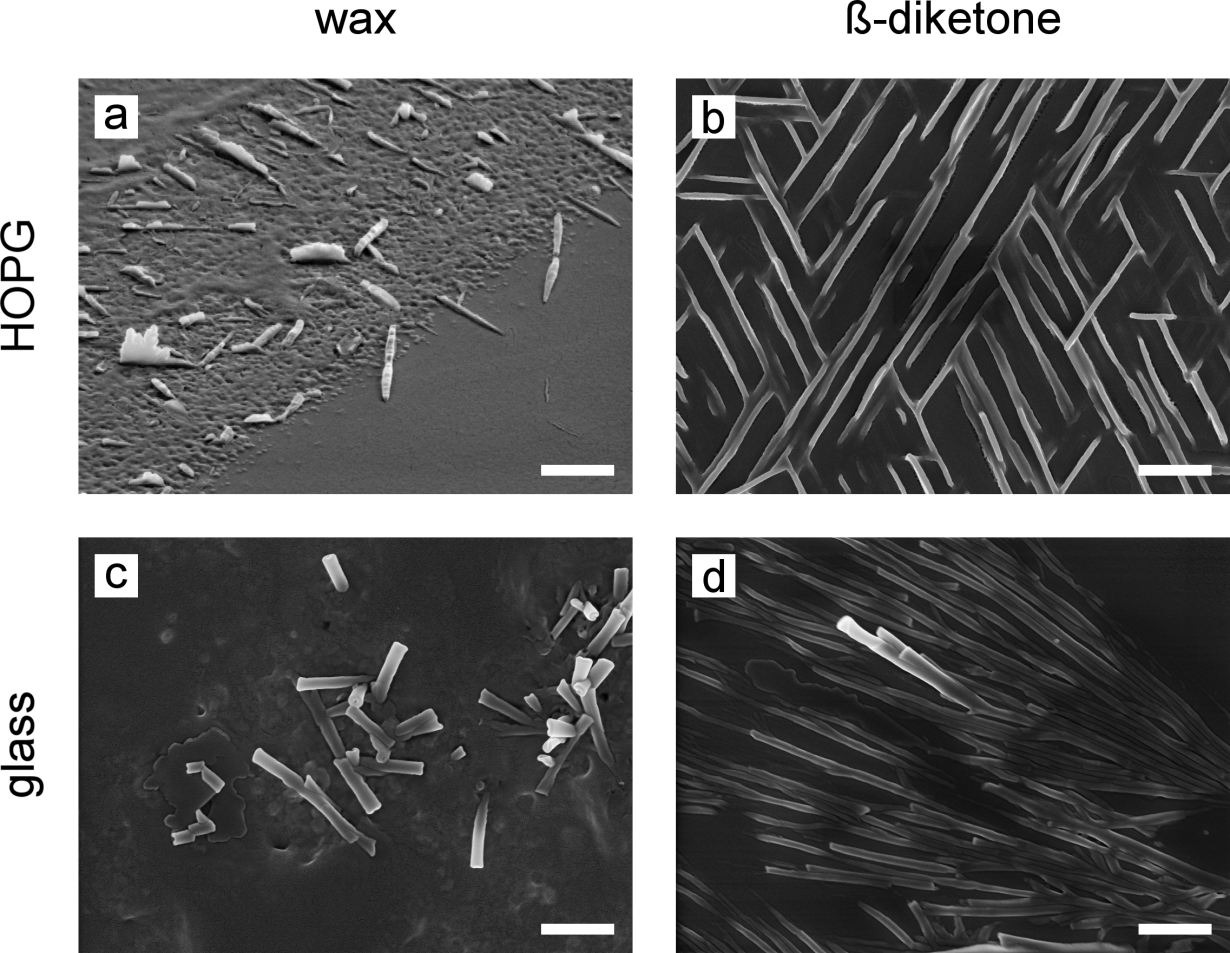
SEM micrographs of wax and ß-diketone recrystallized on HOPG and glass after three days. Left column: wax solution; right column: ß-diketone solution. (a) Tilted SEM micrograph of irregular platelets parallelly oriented in acute angles. (b) Network-like structure made of platelets. (c) Vertically protruding tubules, and (d) horizontally grown tubules. Scale bars: 2 µm.

After evaporation of the chloroform, wax and ß-diketone were irregularly distributed on the substrates. A circular mass deposition, also known as “coffee ring effect” [36], was visible in low magnification. In areas of very high mass accumulation, both substances assembled into plates with a random orientation and into tubules on both substrates. On areas with lower mass accumulation, different structures were found depending on the substrate. Although wax and pure ß-diketone formed tubules on glass and on HOPG, mainly platelets were seen on HOPG (Figure 2). Platelets are similar structures to plates but smaller.

#### Recrystallization on HOPG

Wax mainly formed entire platelets as well as irregular platelets parallelly oriented in acute angles on HOPG (Figure 2a). They stood upright on their longer narrow side as shown in SEM images recorded on tilted samples. In the side view, their crenate, comb-like structure was visible. In between platelets, some single-flat growing tubules were also detected. In areas where platelets were denser, they were connected with each other and formed a structured network (comparable to Figure 2b).

ß-Diketone also formed platelets that were similar in form and orientation to those formed from plant wax. However, entire platelets rather than irregular ones were formed. The platelets built a network-like structure on HOPG (Figure 2b). Tubules and transitional forms between platelets and plates were additionally formed. The tubules grew horizontally on the surface forming a continuous trace with tree-like ramifications (comparable to Figure 2d). On a few spots, single tubules protruded from the base clearly showing their openings.

#### Recrystallization on glass

On glass, wax and ß-diketone formed tubules that differed in their spatial arrangement. In areas where a lot of wax was aggregated, the tubules were inclined upwards out of the wax mass (Figure 2c). On areas with very low mass accumulation, single tubules grew horizontally on the substrate. As observed on HOPG, ß-diketone formed many horizontally grown tubules on glass. They were very close together and a few tubules protruded upwards (Figure 2d). Furthermore, densely arranged plates with tubules in between or growing on the top of them were detected.

### AFM investigation of wax and ß-diketone recrystallization

Recrystallization of wax and pure ß-diketone was investigated by AFM. The measurements started 6 to 14 min after the evaporation of the solvent and continued for 1 to 23 h.

#### Recrystallization on HOPG

**Figure 3:**
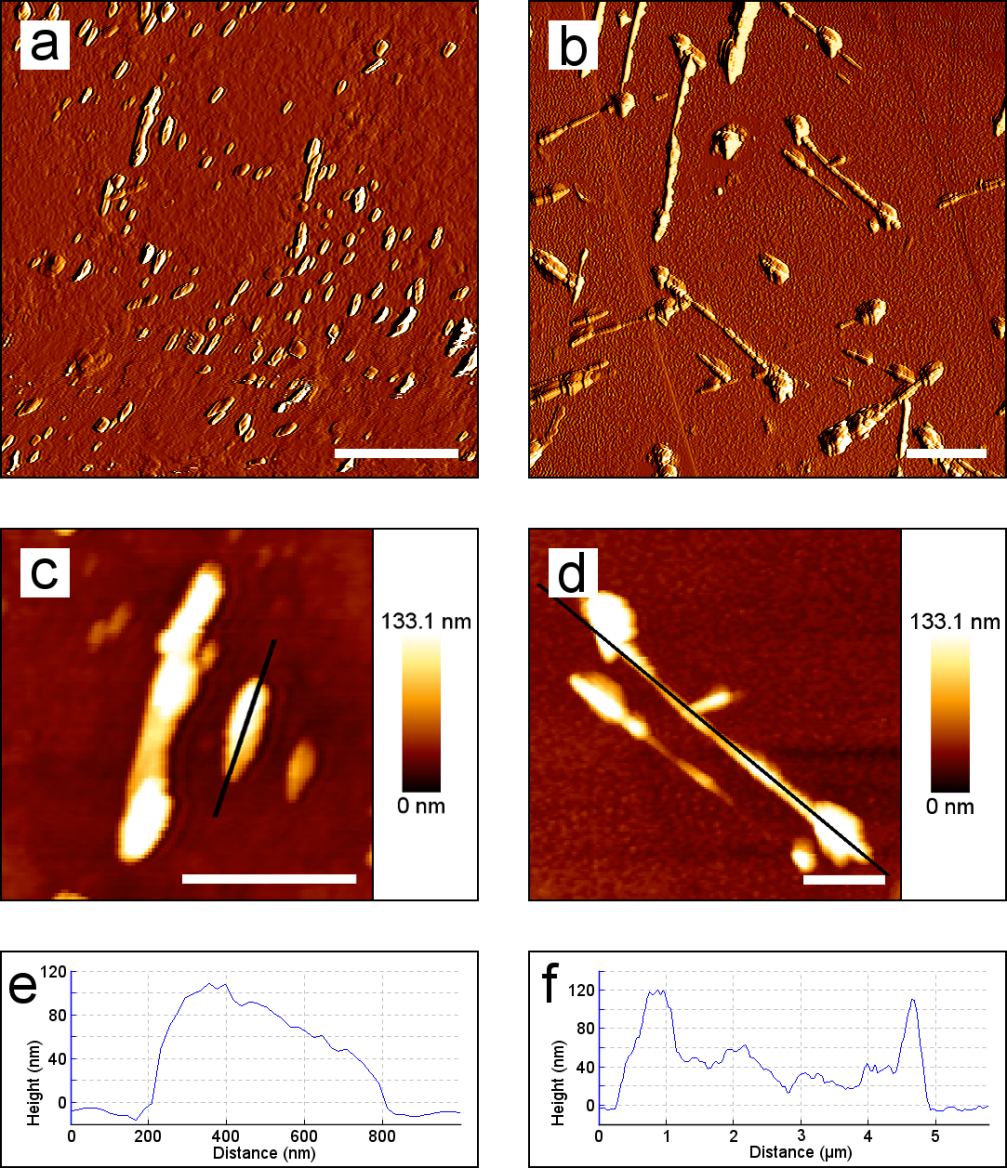
AFM images of recrystallized wax on HOPG. Two kinds of platelets were detected: (a,c,e) shorter platelets with varying shapes of the top edges; (b,d,f) longer platelets with deeply grooved top edges. (a) Platelets arranged in one direction after 50 min. (b) Platelets arranged parallelly or and in acute angles, 4 h and 28 min after the application of the wax solution. (c,d) Detailed view of the platelets with the corresponding longitudinal section analysis (e,f). Scale bars in (a,b): 2.5 µm, in (c,d): 1 µm.

The first AFM images of real-time investigation of wax recrystallization on HOPG were captured 10 to 13 min after the evaporation of the solvent. The AFM examination of wax self-assembly on HOPG showed platelets (Figure 3) as seen in SEM analysis. In AFM micrographs, two types of platelets with varying sizes and upper edges were identified. Firstly, 0.21–1.74 µm long platelets arranged in one direction and irregular top edges were observed (Figure 3a). An analysis of a longitudinal section through the platelets revealed varying shapes of the top edges of these platelets (Figure 3c, Figure 3e). This was also confirmed by tilted SEM images (Figure 2a). The shapes varied between semicircular, oblique and crenate. The maximum heights of these platelets varied between 62.5 and 196 nm. Secondly, some platelets with similar height ranges (41.4–195 nm) but different length and top edges were observed. These platelets were longer (0.5–6 µm) and either stood vertically in a parallel orientation or formed acute angles with each other (Figure 3b). Their upper edges were grooved and thicker on their endings (Figure 3b,d,f).

#### Recrystallization on glass

**Figure 4:**
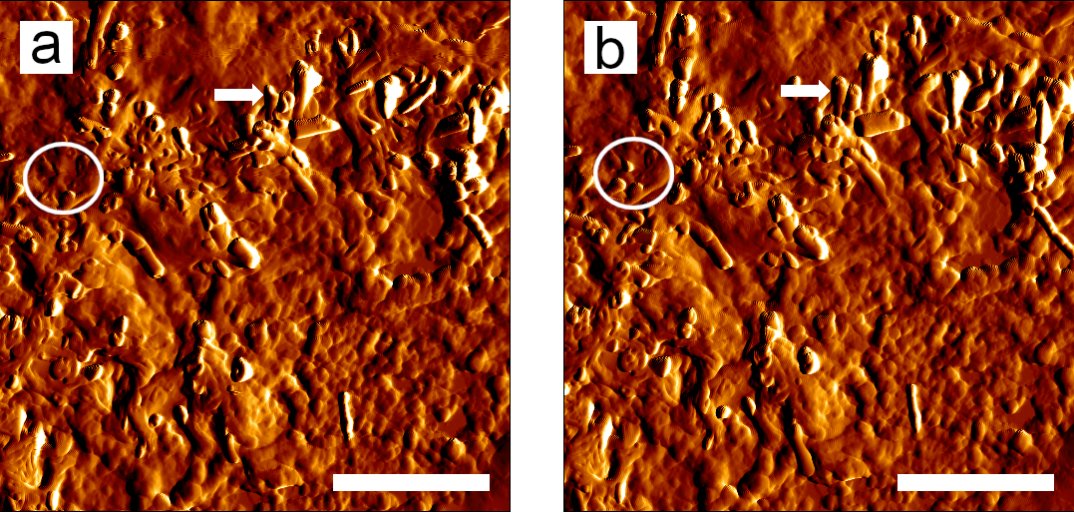
Comparison of AFM images of recrystallized wax on glass (a) 45 and (b) 71 min after the application of the wax solution. The circles indicate the formation and the arrows the elongation of the tubules. Scale bars: 2 µm.

**Figure 5:**
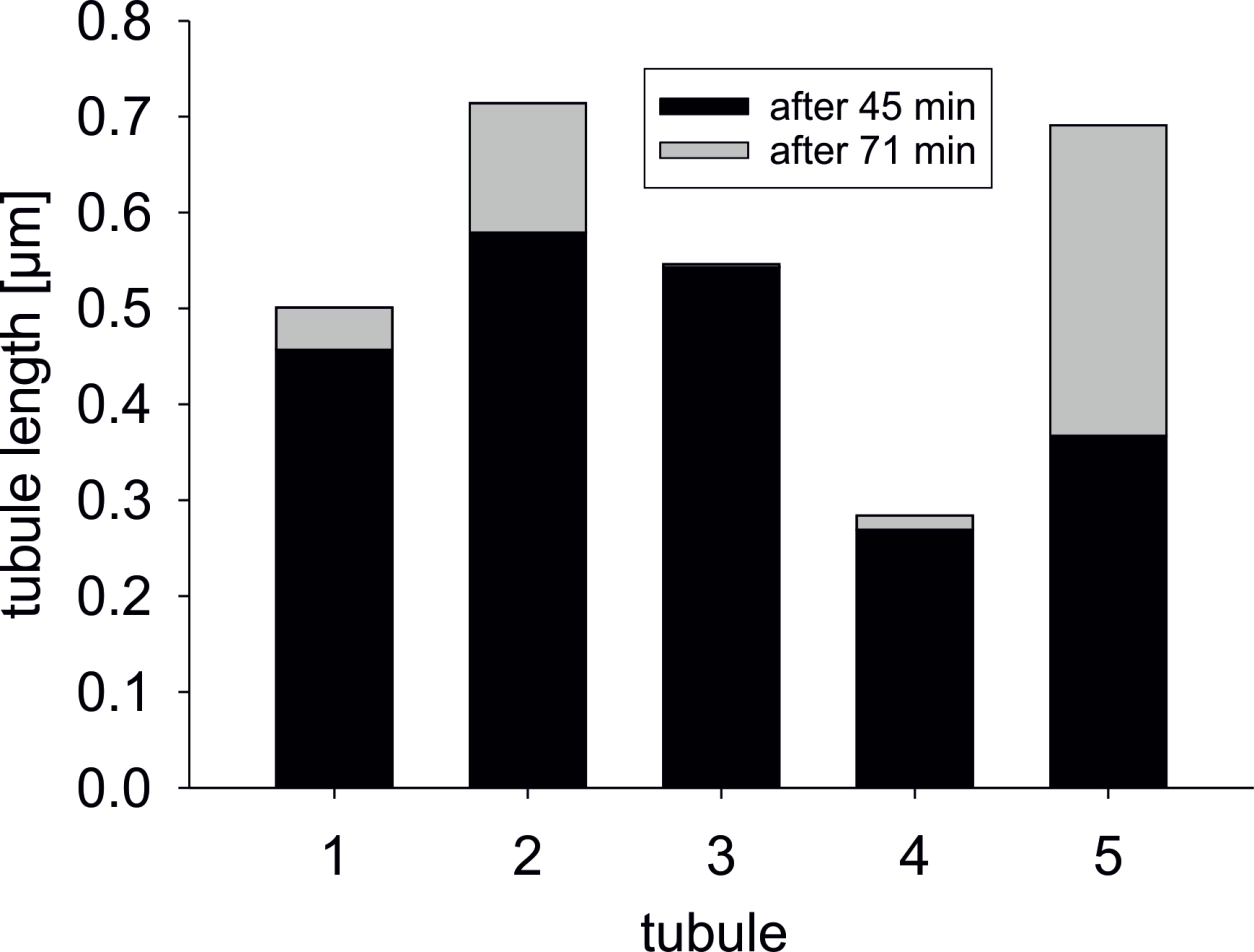
Diagram of length increase of recrystallized tubules (1–5) from a wax solution on glass within 26 min. The measurements of length increase were performed 45 and 71 min after the application of the wax solution on the substrate.

Similar to SEM micrographs, AFM images also showed that a high number of small tubules and some larger, protruding tubules were formed from the wax solution in areas of high mass accumulation (Figure 4). On one sample, a consecutive scanning of such an area showed the formation of new tubules (white circles in Figure 4a and Figure 4b) and an increase in length of already existing tubules (white arrows in Figure 4a and Figure 4b). The length increase of five tubules was measured over 26 min. Within that time, the tubules had a varying length increase from 3 up to 300 nm (Figure 5).

**Figure 6:**
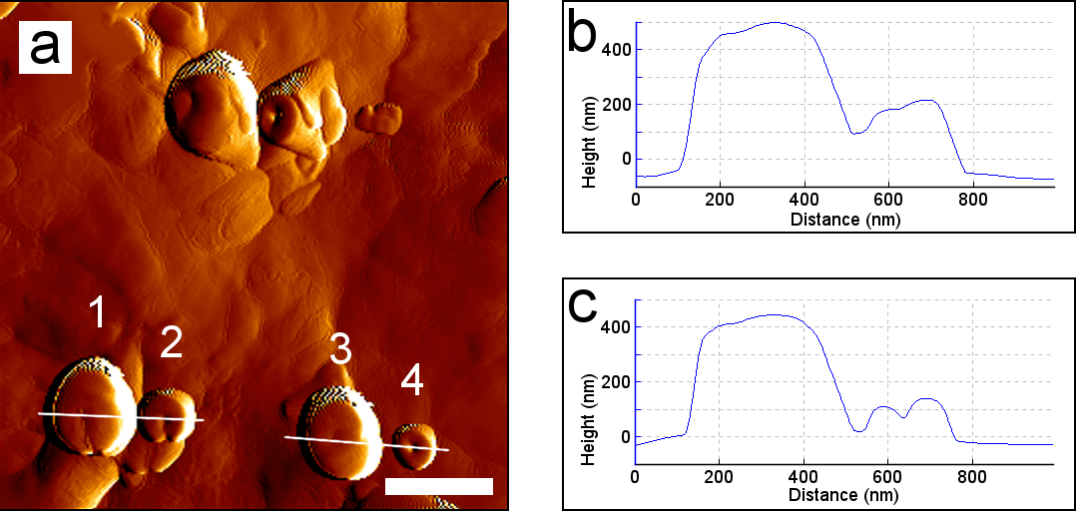
AFM image of recrystallized wax tubules from a wax solution on glass. (a) Top view of vertically growing tubules 22 h and 39 min after application. (b) Cross section of tubule 1 and 2 and (c) cross sections of tubule 3 and 4 seen in (a). Scale bar: 500 nm.

**Figure 7:**
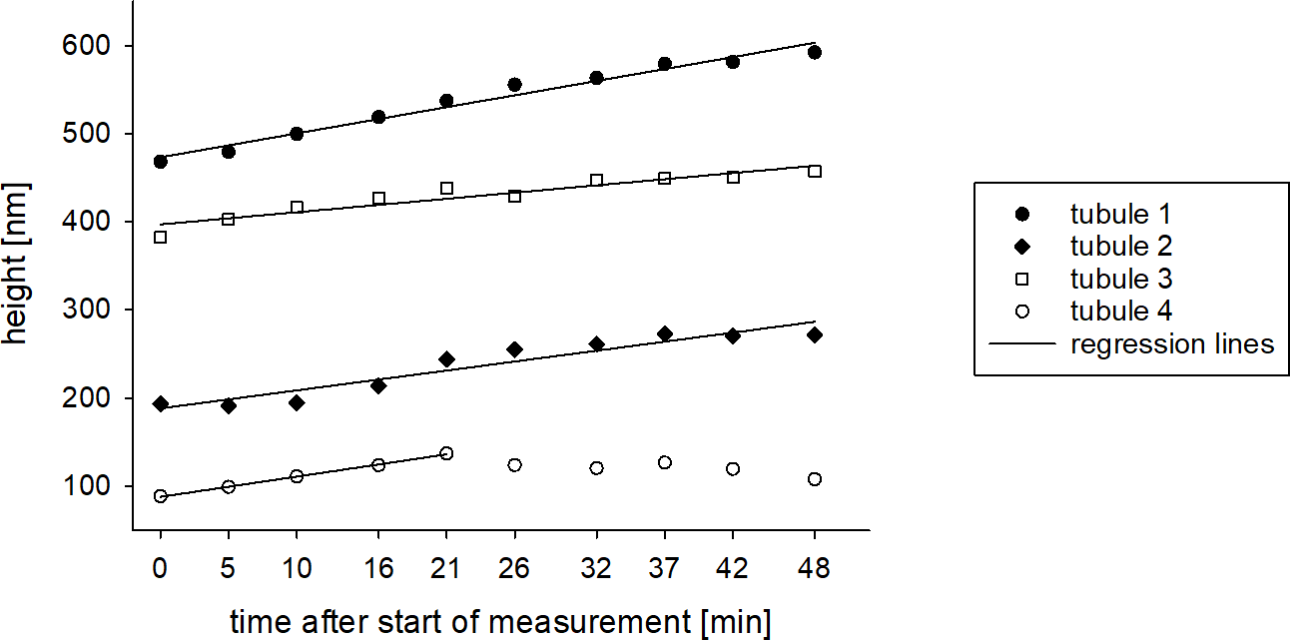
Diagram of height increase of vertically growing tubules from a wax solution on glass. Time zero represents height measurements 22 h and 29 min after the application of the wax solution. The curves were fitted by linear regression lines (f = y_0_ + a·x). R² ≥ 0.9.

New tubules were still generated 22 h and 29 min after the application of the wax solution. The growth of four vertically growing tubules was measured by consecutive scans (Figure 6). One of the tubules (tubule 2, Figure 6) was not completely closed to a cylindrical structure and another tubule showed a step on its top (tubule 4, Figure 6). The tubules showed a height increase of 20 to 40% with growth velocities of 0.3 to 0.5 nm/min (Figure 7). Since tubule 4 only grew until minute 21, the growth rate of tubule 4 was determined only for this period. After that, the height of the tubule decreased.

**Figure 8:**
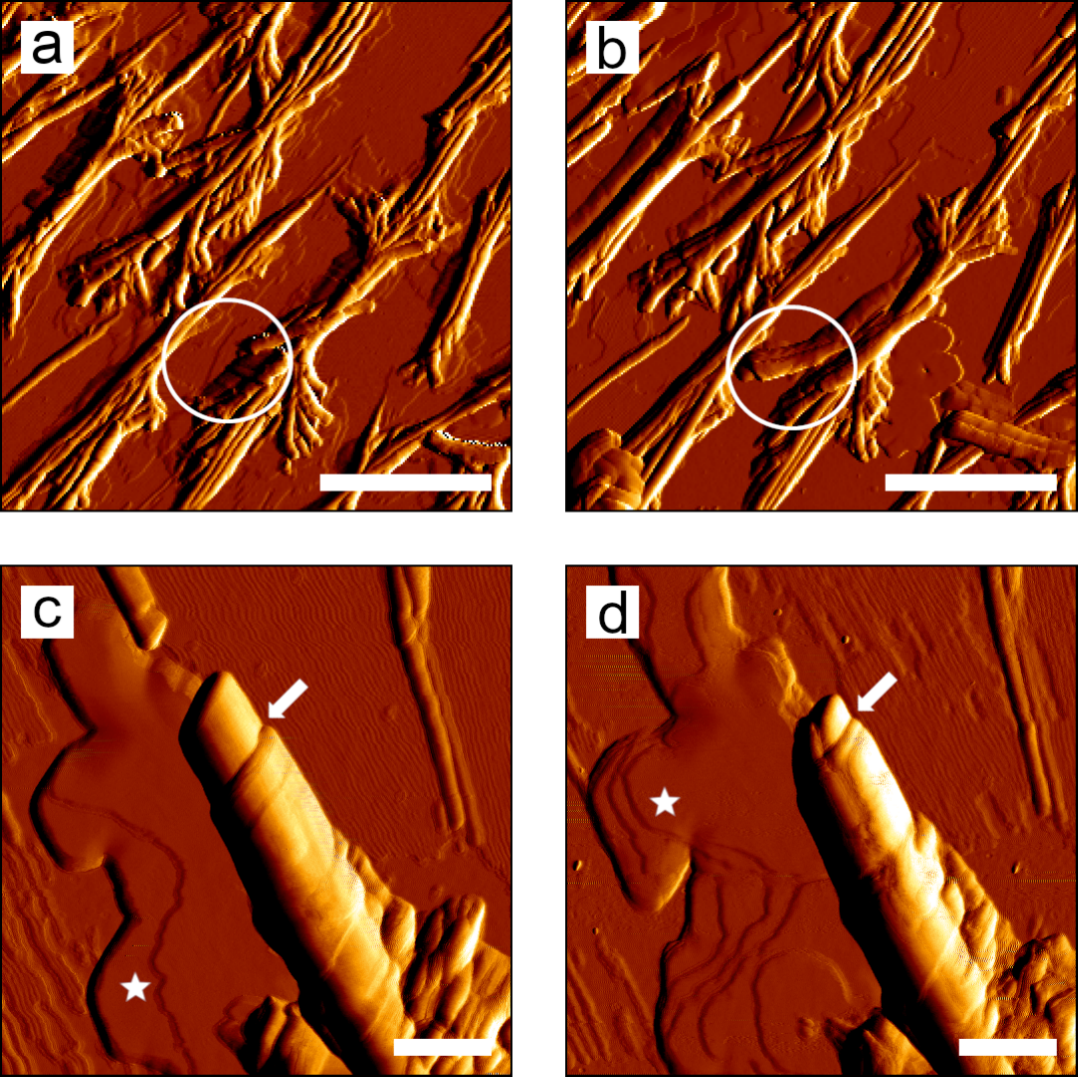
AFM images of recrystallized tubules of a ß-diketone solution on glass. (a,b) Flat-growing branched tubules ordered as traces in the same position (a) 50 min and (b) 4 h and 45 min after the application of the ß-diketone solution. White circles indicate length increase of a tubule and white arrows show an expanding layer close to the tubules. (c,d) Detailed view of a tubule tip with the edge of a layer on its surface (white arrows) and layers with several steps (white stars); (c) 6 h and 8 min and (d) same position, 6 h and 22 min after the application of the ß-diketone solution. Scale bars in (a,b) 2 µm, in (c,d) 500 nm.

The first AFM images of the investigation of ß-diketone recrystallization on glass were taken between 17 and 35 min after the evaporation of the solvent. Tubules with several branches arranged in flat lying-traces, as seen in the SEM analysis (Figure 2d), were also found in the AFM analysis (Figure 8). Consecutive scans of the same area revealed an increase in length of a single tubule from 0.9 to 1.4 µm within 4 h. Step heights of 21 ± 2 nm were measured on the outside of the tubule walls (Figure 8c). Subsequent scans showed that the tubule tip was shortened over time. Additionally, layers with step heights of 3.5 ± 0.3 nm were formed on the substrate (Figure 8c, Figure 8d, indicated by white stars). These layers expanded over time and further layers were formed on top.

## Discussion

Since we see comparable structures in our recrystallization study on an artificial surface and on *E. gunnii* leaves [30], we expect that our experiments help to understand the formation of ß-diketone tubules in nature. On *E. gunnii* leaves, wax regeneration can be observed by AFM, but the measurement is limited to small areas and short times due to the roughness of the leaf surface [30]. In addition, fresh plant leaves containing water are heated by the laser beam during scanning, which can cause a moderate expansion of the sample and thus a drift of the scan area after several scans. Studies of recrystallization on non-biological substrates have the advantage that the substrates are smooth, non-elastic, and have a lower thermal absorption than hydrous biological surfaces when scanned with AFM for extended periods of time. Here, wax and pure ß-diketone dissolved in chloroform were recrystallized on non-polar crystalline HOPG and on polar amorphous glass. Recrystallization on the artificial substrates showed an unequal distribution of the deposited wax and of ß-diketone. Therefore, it took several attempts to find suitable areas to observe the growth of 3D structures. This caused a delay of some minutes to start the observation.

### Distribution of recrystallized structures

Scanning electron microscopy investigations of recrystallized structures showed a circular pattern with areas of greater and lesser amounts of deposited mass. This phenomenon known as “coffee ring effect” is caused by capillary flow of the solvent from the middle of the droplet towards its three-phase (solid–liquid–air) contact line. During evaporation, the molecules are aggregated on the outer edge of the droplet where evaporation is at the highest rate [36–37]. It is reasonable to assume that an increase of concentration at the droplet edges also promotes crystal nucleation in that area. The growing crystals then, in turn, reduce the concentration in the droplet. However, the evaporation of the solvent leads to an increase in concentration again after some time, which in turn leads to crystal nucleation. This process is repeated several times and due to the loss of droplet volume through evaporation, several rings of different diameters with greater mass accumulation were formed. To avoid this unequal mass distribution, recrystallization studies with thermal evaporation of waxes on artificial surfaces were carried out by Niemietz [38]. However, such solvent-free recrystallization of tubules requires storage at 50 °C for a couple of days. For our study we used a fast evaporating solvent, which provides a faster recrystallization due to the solvent-driven mobility of the molecules [25].

### ß-Diketone: the shape-determining compound

For plant waxes mainly composed of primary alcohols, it has already been demonstrated that the main components can determine the crystal shape [15,27]. However, minor components can be shape determining too, as shown for nonacosanol tubules [15,25]. Nonacosanol tubules could only be crystallized with the addition of a specific amount (>2% of mass) of alkandiols [25]. Our results showed that pure ß-diketone alone formed tubules and no additional compound was needed. We, therefore, conclude that in ß-diketone tubules the main component is the shape-determining component. This confirms the study of Meusel et al. [29], in which tubules were also formed by recrystallization of ß-diketones. The positions where the functional groups are located are important for the formation of the tubules. Like in our study, in the study of Meusel the tubules were formed by the recrystallization of ß-diketones substituted at C_14_ and C_16_. However, ß-diketones substituted at positions before C_12_ recrystallized as coiled tubules [29].

### Recrystallized structures on different substrates

A comparison of tubule formation on polar and non-polar substrates was also carried out for nonacosanol tubules [27]. In that study it was shown that the polarity of the substrate had an influence on the formation and the orientation of the tubules: On polar silicon wafers, randomly orientated tubules grew on top of thick crusts, whereas on non-polar HOPG, tubules grew vertically with respect to the substrate. Here, on both substrates, wax and pure ß-diketone crystallized in areas of high mass accumulation into plates and platelets, which were not present on plant leaves. We assumed that the substrates were masked by the deposited masses, thus, the surface polarity had no direct influence on the shape of the crystallized structures. Meusel [39] also showed deviant structures such as plates formed by pure hentriacontane-14,16-dione. There, the polymorphism (different shapes formed by the same chemical compound) of the recrystallized ß-diketone was dependent on the physical conditions (various temperature values and recrystallization velocities).

#### Recrystallization on HOPG

Wax and ß-diketone crystallized on HOPG, mainly as platelets arranged in defined angles. In areas with higher mass accumulation, the platelets were connected with each other and formed a network-like structure (Figure 2b). The orientation of the platelets/network corresponded to the hexagonal structure of the underlying HOPG, indicating a strong influence of the substrate on the shape and orientation of the recrystallized structures. This so-called template effect [40–41] was also described in earlier studies conducted with the primary alcohol octacosan-10-ol [14]. On HOPG some tubules were also formed. In contrast to wax, ß-diketone formed more tubules than wax. Scanning electron microscopy micrographs showed that the tubules grew horizontally with respect to the substrate and were branched in typical acute angles (Figure 2d). The branching of the tubules was also observed by Jetter and Riederer [15] in a recrystallization study of nonacosanol tubules. There, the formation of side branches was explained by the kinetic regime of crystallization, in which the growth of a developing crystal is restricted by the diffusion of the molecules. Thus, new molecules are added mainly at one side of an already existing crystal, resulting in a crystal growth in one direction. In the present experiment, the kinetic regime could be applied after the evaporation of the solvent. Hence, tubule growth occurred preferably in one direction.

#### Recrystallization on glass

On glass mainly tubules were formed by wax and ß-diketone solutions. No transitional forms or helically wound ribbons, as found on leaves, were formed [30]. Although SEM micrographs showed a few isolated tubules, no tubules were detectable by AFM on areas with low mass accumulation on glass. In areas with higher mass accumulation the formation of ß-diketone tubules was observed. The tubules extended at varying speeds, as described in recrystallization studies of *Tropaeolum majus* wax [31]. There, the variation of velocity was associated with differences in the distribution of wax masses on the substrates. In the present study, one tubule shrank after the growth phase (Figure 7), which may also be related to the available wax masses in the vicinity of the tubule. The tubule might have been in the dynamic formation process at that point, during which the wax could also be released into the surrounding area again. The process of secondary alcohol tubule formation was shown by recrystallization of *N. nucifera* and *T. majus* wax on HOPG [27–28]. The formation of secondary alcohol tubules started with a bent rodlet, which then closed to a ring and finally extended to a tubule by the accumulation of new wax. A helical growth mechanism was visible by the movement of a stepped end at the growing tip of the tubule. In our study a ß-diketone tubule showed a similar stepped end at the tip of the tubule, indicating a similar growth mechanism to the helical growth mechanism described for secondary alcohol tubules. The investigations on glass revealed horizontally grown and parallelly oriented branched tubules. These structures were probably formed within a few minutes after solvent evaporation since the tubules were already fully developed when the first AFM image was taken. Atomic force microscopy investigation showed layers enveloping the tubules (approximately 21 nm thick) and a thin layer (with step heights of approximately 3 nm on the substrate next to the tubules, Figure 8c and Figure 8d), which were not detectable by SEM. Consecutive images showed that the tip of the tubule was shortened over time and new steps on existing underlying layers of approximately the same height (3 nm) were grown. It is plausible to assume that the crystal structure of the newly formed wax shapes was not yet stable. After evaporation of the chloroform, some chloroform molecules may have remained in between the recrystallizing molecules. Hence, the molecules were still mobile and partly hindered by the chloroform molecules in their arrangement. Tubule formation from the wax mixture requires a separation of the ß-diketones from the bulk material after solvent evaporation. Such a process could not be observed here for the eucalyptus wax, but was found in earlier studies with native wax mixtures of nonacosanol waxes [42].

## Conclusion

Tubules were recrystallized from *E. gunnii* wax and from a ß-diketone solution on artificial substrates. Both the wax mixture and pure ß-diketone showed similar tubules to those found on leaves. However, deviating platelet-shaped and layered structures not found on plant surfaces were formed, especially on areas with high mass accumulation. The presented data showed that ß-diketone tubules are formed by self-assembly and confirmed that ß-diketone is the shape-determining component. It was shown that crystal formation is influenced by the underlying substrate: on polar glass mainly flat lying tubules were formed, while on non-polar HOPG mainly platelets were formed. This clearly shows that the crystallization process is determined by crystal-forming molecules and by the underlying substrate. It was demonstrated that AFM is a suitable tool to study dynamic processes, even in soft materials such as natural wax. With this technique it was possible to record the elongation of ß-diketone tubules in real time. In the top view of an elongating tubule a stepped end was detected. However, it is not yet clear whether ß-diketone tubules have a similar or different growth mechanism than that of helically elongated nonacocosan-10-ol tubules. At this early stage of studying the formation of ß-diketone tubules, we cannot yet provide a molecular model of ß-diketone tubules, which explains the observed differences caused by the substrate polarity in this current study. In future studies, the molecular arrangement of the observed layers and tubules could provide a deeper understanding of the architecture and formation of these protective surface structures frequently found in plants.
